# Considering the design effect in cluster sampling

**DOI:** 10.15171/jcvtr.2019.14

**Published:** 2019-02-17

**Authors:** Yousef Alimohamadi, Mojtaba Sepandi

**Affiliations:** ^1^Pars Advanced and Minimally Invasive Medical Manners Research Center, Pars Hospital, Iran University of Medical Sciences, Tehran, Iran; ^2^Department of Epidemiology and Biostatistics, School of Public Health, Tehran University of Medical Sciences, Tehran, Iran; ^3^Health Research Center, Lifestyle Institute, Baqiyatallah University of Medical Sciences, Tehran, Iran; ^4^Department of Epidemiology and Biostatistics, Faculty of Health, Baqiyatallah University of Medical Sciences, Tehran, Iran

## Dear Editor,

We read the valuable manuscript with the title: *Association of modified Nordic diet with cardiovascular risk factors among type 2 diabetes patients: a cross-sectional study* that published in *J Cardiovasc Thorac Res*.^1^ In this manuscript authors have said “cluster random sampling methods were used to select participants. Throughout the five sectors of Isfahan, we randomly chose two centers (clinics) from each area. Based on previously calculated mean and standard deviations for BMI in this population, our target number of participants was 143”.

We have some points about sampling method and sample size determination in mentioned manuscript. When cluster sampling is used the effect of intra-cluster correlation (ICC, or the strength of correlation within clusters) must be regarded for sample size calculation.^2^ This effect called the design effect (Deff). It is a correction factor that is used to adjust the required sample size for cluster sampling. The required sample size, assuming a simple random sample (SRS), should be calculated, and then multiplied by the Deff.^3,4^

Deff represents the degree of variance inflation that attributes to cluster sampling.^5^ The value of Deff for prevalence estimation in a cross-sectional study depends on the average number of subjects sampled per cluster (n) as well as ICC^3^:

Deff= 1 + p (n – 1)

p=ICC

Suppose in a cross-sectional study, SRS is used to estimate the mean of a parameter. The variance of the sample mean is calculated as follows:

$$V_{SRS}(\bar{Y})=\sigma^{2}/n$$

If instead of SRS, a cluster design is used with k clusters and m subject in each cluster, the variance is calculated as follows^5^:

$$V_{Cluster}(\bar{Y})=(\sigma^{2}/k*m)*[1+p(m-1)]$$

The expression [1 + p (*m* − 1)] in the above formula is called the variance inflation factor (VIF). So in cross-sectional studies when the unit of sampling is a cluster, but the sample size is determined according to the SRS assumption, the determined sample size should be multiplied by Deff or VIF^3^:

Deff= ^Variance^_ Cluster_**/**^variance^_ SRS_

n = n_SRS_∗ Deff

## Ethical issues

Not applicable.

## Competing interests

Authors declare no conflict of interest in this study.
